# Nurse versus physician-provision of early medical abortion in Mexico: a randomized controlled non-inferiority trial

**DOI:** 10.2471/BLT.14.143990

**Published:** 2015-02-19

**Authors:** Claudia Diaz Olavarrieta, Bela Ganatra, Annik Sorhaindo, Tahilin S Karver, Armando Seuc, Aremis Villalobos, Sandra G García, Martha Pérez, Manuel Bousieguez, Patricio Sanhueza

**Affiliations:** a Instituto Nacional de Salud Pública, Septima Cerrada de Fray Pedro de Gante No 50, Col Seccion XVI, Tlalpan, Mexico City, 14000, Mexico.; b Department of Reproductive Health and Research, World Health Organization, Geneva, Switzerland.; c Population Council Mexico, Mexico City, Mexico.; d Mexico City Ministry of Health, Reproductive Health Program, Mexico City, Mexico.

## Abstract

**Objective:**

To examine the effectiveness, safety, and acceptability of nurse provision of early medical abortion compared to physicians at three facilities in Mexico City.

**Methods:**

We conducted a randomized non-inferiority trial on the provision of medical abortion and contraceptive counselling by physicians or nurses. The participants were pregnant women seeking abortion at a gestational duration of 70 days or less. The medical abortion regimen was 200 mg of oral mifepristone taken on-site followed by 800 μg of misoprostol self–administered buccally at home 24 hours later. Women were instructed to return to the clinic for follow-up 7–15 days later. We did an intention-to-treat analysis for risk differences between physicians’ and nurses’ provision for completion and the need for surgical intervention.

**Findings:**

Of 1017 eligible women, 884 women were included in the intention-to-treat analysis, 450 in the physician-provision arm and 434 in the nurse-provision arm. Women who completed medical abortion, without the need for surgical intervention, were 98.4% (443/450) for physicians’ provision and 97.9% (425/434) for nurses’ provision. The risk difference between the group was 0.5% (95% confidence interval, CI: −1.2% to 2.3%). There were no differences between providers for examined gestational duration or women’s contraceptive method uptake. Both types of providers were rated by the women as highly acceptable.

**Conclusion:**

Nurses’ provision of medical abortion is as safe, acceptable and effective as provision by physicians in this setting. Authorizing nurses to provide medical abortion can help to meet the demand for safe abortion services.

## ملخص

**الغرض:**

دراسة فعالية ومأمونية ومقبولية تقديم خدمات الإجهاض الطبي المبكر بواسطة الممرضات مقارنة بالأطباء في ثلاثة مرافق في مكسيكو سيتي.

**الطريقة:**

أجرينا تجربة غير دونية عشوائية بشأن تقديم خدمات الإجهاض الطبي والاستشارات حول وسائل منع الحمل بواسطة الأطباء أو الممرضات. وكانت المشاركات نساءً حوامل يلتمسن الإجهاض في مدة حملهن التي بلغت 70 يوماً أو أقل. وكان نظام الإجهاض الطبي يتكون من 200 ملغم من ميفيبريستون الفموي الذي يتم تناوله في المرفق ثم 800 ميكروغرام من ميزوبروستول الذي تتناوله المشاركة في الدراسة بنفسها عن طريق الفم في المنزل بعدها بأربع وعشرين ساعة. وتم إرشاد النساء إلى العودة إلى العيادة للمتابعة لمدة تتراوح من 7 إلى 15 يوماً بعدها. وأجرينا تحليل نية العلاج لفروق الاختطار بين تقديم هذه الخدمات بواسطة الأطباء والممرضات بخصوص الاستكمال والحاجة إلى التدخل الجراحي.

**النتائج:**

تم إدراج 884 امرأة، من أصل 1017 امرأة مؤهلة، في تحليل نية العلاج وإدراج 450 امرأة في مجموعة تقديم هذه الخدمات بواسطة الأطباء و434 امرأة في مجموعة تقديم هذه الخدمات بواسطة الممرضات. وكانت نسبة النساء اللاتي استكملن الإجهاض الطبي، دون الحاجة إلى التدخل الجراحي 98.4 % (443/450 امرأة) عند تقديم هذه الخدمات بواسطة الأطباء و97.9 % (425/434 امرأة) عند تقديم هذه الخدمات بواسطة الممرضات. وكان فرق الاختطار بين المجموعة 0.5 % (فاصل الثقة 95 %: من -1.2 % إلى 2.3 %). ولم توجد فروق بين مقدمي هذه الخدمات بالنسبة لمدة الحمل التي تم دراستها أو استعمال وسائل منع الحمل لدى النساء. وقيّمت النساء كلا نوعي مقدمي هذه الخدمات بوصفهما مقبولين بدرجة عالية.

**الاستنتاج:**

تقديم خدمات الإجهاض الطبي بواسطة الممرضات مأمون ومقبول وفعال بنفس قدر مأمونية ومقبولية وفعالية تقديم هذه الخدمات بواسطة الأطباء في هذه البيئة. ومن الممكن أن يساعد السماح للمرضات بتقديم خدمات الإجهاض الطبي في تلبية الطلب على خدمات الإجهاض المأمونة.

## 摘要

**目的:**

对比调查墨西哥城三个医疗设施的护士和医生提供的早期医疗堕胎服务的有效性、安全性和可接受性。

**方法:**

我们对医生或护士提供的药物堕胎和避孕咨询服务进行了随机非劣效试验。参与者是寻求堕胎的70 天或更短妊娠期孕妇。药物堕胎方案是现场服用200毫克口服米非司酮，接着是24小时之后在家口服800微克的米索前列醇。指导孕妇在后面的7–15天后回到医院接受检查。我们对医生和护士提供的完成堕胎服务之间的风险差异和外科干预的必要性执行意向性治疗分析。

**结果:**

在1017名合格的女性中，有884名女性参与意向性治疗分析，其中医生服务组有450名，护士服务组有434名。完成药物堕胎且无需手术治疗的女性，医生服务组占98.4%（443/450），护士服务组占97.9%（425/434）。组之间的风险差异为0.5%（95%置信区间：-1.2%至2.3%）。经调查的妊娠期持续时间或使用避孕用具方法的提供者之间没有差别。将这两种类型的提供者均被女性评为高度可接受。

**结论:**

在此设置中，护士和医生提供的药物堕胎服务具有同样的安全性、可接受性和有效性。授权护士提供药物堕胎服务有助于满足安全堕胎服务的需求。

## Résumé

**Objectif:**

Examiner l'efficacité, la sécurité et l'acceptabilité de l'avortement médical précoce pratiqué par un(e) infirmier(ière) par rapport au même acte réalisé par un médecin dans trois établissements de la ville de Mexico.

**Méthodes:**

Nous avons mené un essai de non-infériorité randomisé sur l'avortement médical et le conseil en matière de contraception pratiqués par des médecins ou des infirmiers. Les participantes étaient des femmes enceintes souhaitant avorter à un terme de grossesse de 70 jours ou moins. Le protocole de l'avortement médical était de 200 mg de mifépristone administrés sur place par voie orale, suivis de 800 μg de misoprostol auto-administrés par voie orale à la maison 24 heures plus tard. Les femmes avaient reçu comme consigne de revenir à la clinique pour y être suivies 7 à 15 jours plus tard. Nous avons effectué une analyse en intention de traiter pour les différences de risque entre l'avortement médical pratiqué par les médecins et par les infirmiers et le besoin d'une intervention chirurgicale.

**Résultats:**

Parmi les 1 017 femmes éligibles, 884 femmes étaient incluses dans l'analyse en intention de traiter, 450 dans l'acte d'avortement pratiqué par un médecin et 434 dans l'acte d'avortement pratiqué par un(e) infirmier(ière). Les femmes qui ont subi un avortement médical sans avoir eu besoin d'une intervention chirurgicale étaient de 98,4% (443/450) pour l'avortement pratiqué par un médecin et de 97,9% (425/434) pour l'avortement pratiqué par un(e) infirmier(ière). La différence de risque entre les groupes était de 0,5% (intervalle de confiance à 95%: de 1,2% à 2,3%). Il n'y avait pas de différence entre les professionnels de santé pour les termes de la grossesse examinés ou la prise de méthode contraceptive des femmes. Par ailleurs, les femmes acceptaient complètement les deux types de professionnels de santé.

**Conclusion:**

Dans ce contexte, l'avortement médical pratiqué par les infirmiers est aussi sûr, acceptable et efficace que celui pratiqué par les médecins. Autoriser les infirmiers à pratiquer l'avortement médical peut aider à répondre à la demande en matière de services d'avortement sécurisés.

## Резюме

**Цель:**

Изучить эффективность, безопасность и переносимость раннего медикаментозного аборта, выполняемого фельдшерами в сравнении с акушерами, в трех учреждениях Мехико.

**Методы:**

Было проведено рандомизированное исследование медикаментозных абортов и консультаций по контрацепции, выполняемых и предоставляемых фельдшерами на предмет их не меньшей эффективности по сравнению с выполнением данных абортов акушерами. В исследовании принимали участие беременные женщины, желающие сделать аборт на сроке беременности до 70 дней. Медикаментозный аборт заключался в приеме в исследовательском центре 200 мг перорального мифепристона с последующим приемом дома 800 мкг буккального мизопростола через 24 часа. Женщины были проинформированы о необходимости посещения клиники через 7-15 дней для проведения последующего наблюдения. Был проведен статистический анализ всех рандомизированных пациентов для определения разности рисков проведения медикаментозного аборта акушерами в сравнении с фельдшерами и необходимости хирургического вмешательства.

**Результаты:**

Из 1017 удовлетворяющих критериям включения женщин 884 были включены в статистический анализ, 450 – в группу выполнения аборта акушерами и 434 – в группу выполнения аборта фельдшерами. Согласно результатам исследования 98,4% (443/450) составляли женщины, которым медикаментозный аборт был выполнен акушерами без необходимости проведения хирургического вмешательства, и 97,9% (425/434) – женщины, которым медикаментозный аборт был выполнен фельдшерами. Разность рисков между группами составляла 0,5% (95%-ный доверительный интервал: −1,2-2,3%). Не были выявлены различия между исполнителями абортов в исследованных сроках беременности или методах контрацепции женщин. Оба типа исполнителей абортов были оценены женщинами как "в высшей степени приемлемыми".

**Вывод:**

В данных условиях медикаментозный аборт, выполненный фельдшерами, является столь же безопасным, допустимым и эффективным, как и аборт, выполненный акушерами. Допуск фельдшеров к выполнению медикаментозных абортов может помочь удовлетворить потребности в услугах по безопасному проведению абортов.

## Resumen

**Objetivo:**

Examinar la eficacia, seguridad y aceptabilidad de la actuación de los enfermeros en los abortos con medicamentos en fases tempranas de la gestación en tres centros de Ciudad de México en comparación con la actuación los médicos.

**Métodos:**

Se llevó a cabo un ensayo de no inferioridad aleatorio sobre la realización de abortos médicos y asesoramiento anticonceptivo por parte de médicos o enfermeros. Las participantes fueron mujeres embarazadas que buscaban interrumpir una gestación de 70 días o menos. El régimen para el aborto con medicamentos consistió en 200 mg de mifepristona por vía oral, administrados en el centro, seguidos de 800 μg de misoprostol autoadministrados por vía oral en casa 24 horas más tarde. Se indicó a las mujeres que acudieran a la clínica para realizar un seguimiento entre 7 y 15 días más tarde. Se realizó un análisis por intención de tratar de las diferencias de riesgo entre la asistencia de los médicos y los enfermeros para el término y la necesidad de una intervención quirúrgica.

**Resultados:**

De 1017 mujeres elegibles, se incluyeron 884 en el análisis por intención de tratar, 450 en el brazo atendido por médicos y 434 en el grupo atendido por enfermeros. De las mujeres que se sometieron al aborto con medicamentos, el 98,4 % (443/450) de las atendidas por médicos y el 97,9 % (425/434) de las atendidas por enfermeras no requirieron ninguna intervención quirúrgica. La diferencia de riesgo entre el grupo fue de 0,5 % (intervalo de confianza del 95 %: −1,2 % al 2,3 %). No hubo diferencias entre los proveedores para la duración de la gestación examinada o método anticonceptivo de las mujeres. Las mujeres calificaron ambos proveedores como muy aceptables.

**Conclusión:**

Los enfermeros son tan seguros, aceptables y eficaces como los médicos a la hora de asistir un aborto con medicamentos. Autorizar a las enfermeras a realizar abortos con medicamentos puede ayudar a satisfacer la demanda de servicios para un aborto seguro.

## Introduction

In Mexico City, early elective abortion to terminate pregnancy was legalized in April 2007.^1^^–^^3^ Up to June 2013, 100 000 abortions have been provided by trained physicians in Ministry of Health hospitals and clinics.^4^ In 2009, it was estimated that 13.5% (16 475/122 455) of all abortions in Mexico City were safe and legal and provided by the public sector.^5^ Even though some safe and legal abortions are done in private facilities,^6^ most abortions are done outside sanctioned facilities and are potentially unsafe.^7^ Despite Mexico City’s efforts to expand services and increase the availability of the mifepristone-misoprostol regimen for medical abortion, patient demand is outpacing service capacity. Furthermore, conscientious objections from trained physicians^8^ have further restricted service capacity.

To expand the capacity of the health workforce, task-shifting has been proven to be an important strategy.^9^ Evidence from low-resource settings suggests that trained, mid-level providers can administer medical abortion with similar outcomes as physicians.^10^^–^^14^

In Mexico, nurses tend to have a subordinate role compared to physicians and have traditionally been excluded from decision-making.^15^ Approximately one-third of the Mexico City Ministry of Health personnel are physicians and one-third are nurses.^16^ Authorizing nurses to provide medical abortion could increase the potential capacity for provision of this service and help to address increasing demand.

We aimed to assess the effectiveness, safety and acceptability of nurses’ versus physicians’ provision of early medical abortion in facilities of the Mexico City Ministry of Health.

## Methods

We conducted a randomized controlled non-inferiority trial between November 2012 and January 2013 at two Mexico City Ministry of Health abortion clinics and one hospital. Mean numbers of both medical and surgical abortions performed weekly are 137 and 27 at the clinics and 48 at the hospital, representing 50% of legal abortion provision in Mexico City, a city of 8 851 080 people.^17^

We assumed that physicians and nurses would achieve a 95% completion rate for medical abortions, based on a previous randomized controlled trial^11^ and a meta-analysis on medical abortion efficacy which found successful medical abortion rates using the mifepristone-misoprostol regimen between 91% to 96% depending on gestational duration.^18^ We assumed a 5% non-inferiority margin based on cost-effectiveness and clinical differences – such as the ability of nurses and physicians to determine gestational duration, screen for early medical abortion and determine incomplete abortion.^19^ We used the PASS software version 11 (NCSS, LCC, Kaysville, United States of America) to determine that a sample size of 800 (400 per arm) would be sufficient to detect non-inferiority with 90% power and a one-sided significance level of 0.025, assuming 15% loss to follow-up per arm.

The study protocol was approved by the Mexico City Ministry of Health and Mexico’s National Institute of Public Health institutional review boards. Ethical approval was obtained from the World Health Organization’s (WHO’s) ethics review committee. We invited experts in medical abortion, nursing, ultrasound training and health systems to participate in a scientific advisory and data safety monitoring committee.

The Mexico City Ministry of Health granted temporary permission for nurses to administer medical abortion for study purposes and providers were recruited from existing Ministry of Health personnel. We only recruited physicians who had recently joined clinic staff and who had never provided medical abortion or had only previously managed medical abortion under supervision to minimize unfair comparison between physicians with previous experience and nurses with no experience.

Physicians and nurses received separately one and a half weeks of training on medical abortion management. The training was provided by a certified ultrasonographer and one of the authors. To reach a professional level of ultrasound skill,^20^^,^^21^ all physicians and nurses received 20 hours of abdominal and transvaginal ultrasound training using a Hitachi SSD-3500SX console.^22^ The providers were certified by experienced obstetricians to have achieved required competency. At each site, an experienced obstetrician – not part of the study – was made available for providers to consult as needed.

Women visiting the facilities for an abortion were shown to a private space and screened for eligibility by a nurse participating in the study. Women were invited to participate if they: wanted a medical abortion, were aged 18 years or older, reported a last menstrual period of less than 70 days previously and were willing to provide contact information for follow-up. They were excluded if they had a history of allergy to mifepristone or misoprostol, chronic systemic corticosteroid use, chronic adrenal failure, coagulopathy or current therapy with anticoagulants, inherited porphyria, chronic medical conditions including pre-existing heart, severe hepatic or renal disease and severe anaemia. They were also excluded if they had previously received a medical abortion as part of the Mexico City legal abortion programme. All women were given an opportunity to ask questions before providing written consent. Participants could voluntarily withdraw from the study at any time and for any reason without change to the care they received. Before randomization, enrolled participants provided contact and sociodemographic information via a structured interview.

We generated a list of consecutive identification numbers and randomly allocated a physician or a nurse that should provide medical abortion to each number using R 3.1.2 software for Windows (R Foundation for Statistical Computing, Vienna, Austria). As women were recruited, they were assigned an identification number. Allocation was concealed in a sealed envelope, which was only opened once the participant was considered eligible and consented to enrol.

All enrolled women received clinical care and medical abortion at their first visit. This included a vaginal and pelvic exam, and an abdominal ultrasound to confirm intrauterine pregnancy and gestational duration. According to Mexico City Ministry of Health guidelines, providers must confirm gestational duration via abdominal ultrasound. Providers followed the guidelines’ standard of care regarding ultrasound image interpretation.^22^ Women with an inserted intrauterine device (IUD) that could not be removed before administering mifepristone were excluded from the study. If providers could not confirm gestational duration or intrauterine pregnancy, women were referred to another facility for a β-hCG (human chorionic gonadotropin) fraction test. Since we could not ensure their return to the study, they were excluded.

We used the medical abortion regimen recommended by Mexico City Ministry of Health,^23^ which differs from WHO’s recommendation.^24^ Pregnant women with a gestational duration determined as less than 70 days were given 200 mg of oral mifepristone under supervision followed by instructions to self–administer four tablets of misoprostol (200 μg each) buccally at home, 24 hours later.

All women received misoprostol, instructions for administration and contraceptive method counselling from their assigned provider. The study followed the standard of care in counselling and providers offered a mix of different contraceptive methods. Participants were also given an instruction card for contacting a study representative in the event of any questions or concerns and a pamphlet explaining expected side-effects and symptoms that may warrant prompt medical attention – such as heavy bleeding, fever, headache and abnormal vaginal discharge. Women were instructed to return to the clinic for follow-up 7–15 days later.

At the follow-up visit, providers confirmed completed abortion based on a clinical symptoms checklist, bleeding history and ultrasound results. If the provider determined that the woman had an ongoing pregnancy or incomplete abortion –such as continued bleeding, tissue residue or cramps – participants were offered an additional 800 μg of misoprostol administered at the clinic or hospital, according to Mexico City Ministry of Health practice.^25^ If women requested a manual vacuum aspiration or the provider felt it was warranted – due to remaining fetal tissue, persistent gestational sac or continuation of pregnancy – it was provided on-site by an obstetrician who was not part of the study. Participants who chose to take an additional 800 μg of misoprostol were instructed to return in 7–15 days. If these women still did not have a complete abortion at the second follow-up, a vacuum aspiration was performed on-site, that day.

During follow-up, providers asked participants if they had chosen a post-abortion contraceptive method based on the previous counselling. If available, the method was provided; if not, information on where to obtain it was given.

All adverse and serious adverse events were recorded by providers using a review form that had been reviewed by the scientific advisory and data safety monitoring committee. Events were recorded and analysed to allow for safety reporting.

To assess if there were any significant differences between the two study arms, student’s *t*-tests and *χ*^2^ tests for two independent samples were used. Non-inferiority was tested using intention-to-treat and per-protocol analysis. A 95% confidence interval (CI) for the difference between the physicians’ and nurses’ groups in completed abortion rates between study arms was computed and non-inferiority was accepted if this interval lay completely on the left of the non-inferiority 5% margin, that is, if the difference falls within the predefined equivalence range of 5%. Homogeneity of the three study sites was assessed using the Higgins & Thompson index *H*. Stratified analysis for potential site effects was not conducted because the complete abortion rate at one site was 100% for both study groups.

We conducted a sensitivity analysis to test two different outcome scenarios for women lost to follow-up. Based on observed success rates, we assumed a 100% success rate for women lost to follow-up in both scenarios in the physicians’ group. For the nurses’ group we assumed 78.6% and 71.4% success rates in the first and second scenario, respectively.

Once a complete abortion was confirmed and women were given a contraceptive method or information, a study coordinator not linked to clinical care administered a satisfaction survey that the participants completed on-site. An acceptability scale was constructed using responses to the 14 questions. Some answers were binary – yes or no – and others were categorical – e.g. range of satisfaction and expectation levels. We scored the responses for each question using a range between 0 to 1, where the responses with the lowest acceptability received 0 points and responses with the highest acceptability received 1. The points generated for each question were summed to create a provider acceptability score per participant ranging from 0–14. The mean overall acceptability scores for each provider group were compared to determine women’s satisfaction.

All analyses were conducted using SPSS version 18.0 (SPSS Inc., Chicago, USA).^26^

## Results

Fifteen providers, seven nurses and eight physicians, participated in the study. There was one male nurse and five male physicians. There was no significant difference in homogeneity between the different study sites (*P* = 0.07).

**Fig. 1:**
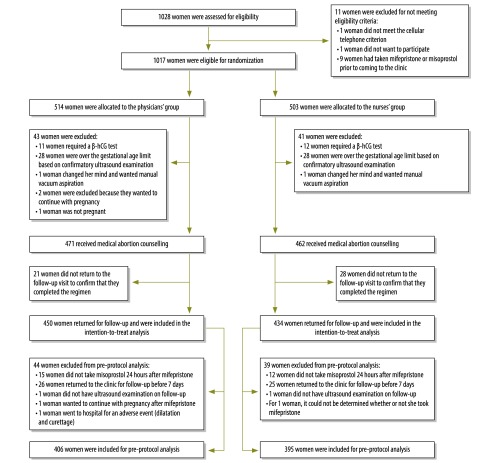
Randomized clinical trial profile of women undergoing first-trimester medical abortion provided by nurses or physicians in Mexico City, 2012–2013

Of 1028 women approached, eleven were excluded for not meeting eligibility criteria (Fig. 1). Of the eligible women, 503 were randomized to receive medical abortion from nurses and 514 women from physicians. Women in both groups had similar characteristics (Table 1).

hCG: human chorionic gonadotropin.

**Table 1:** Characteristics of women undergoing medical abortion provided by nurses or physicians in Mexico City, 2012–2013

Characteristic	No. (%)		
	Physicians’ group (*n* = 514)	Nurses’ group (*n* = 503)	Total (*n* = 1017)
**Age in years, mean (SD)**	25.7 (6.0)	26.3 (6.3)	26.0 (6.2)
≤ 19	60 (11.7)	51 (10.1)	111 (10.9)
20–29	337 (65.6)	312 (62.0)	649 (63.8)
30–39	99 (19.3)	119 (23.7)	218 (21.4)
≥ 40	18 (3.5)	21 (4.2)	39 (3.8)
**Marital Status**			
Single	276 (53.7)	266 (52.9)	542 (53.3)
Married or cohabiting	208 (40.5)	203 (40.4)	411 (40.4)
Separated, divorced, widowed	30 (5.8)	34 (6.8)	64 (6.3)
**Education**			
No education	0 (0.0)	2 (0.4)	2 (0.2)
At least some primary	38 (7.4)	27 (5.4)	65 (6.4)
At least some junior high school	140 (27.2)	142 (28.2)	282 (27.7)
At least some high school or technical school	204 (39.7)	195 (38.8)	399 (39.2)
At least some university	132 (25.7)	137 (27.2)	269 (26.5)
**Occupation^a^**			
Student	122 (23.7)	127 (25.3)	249 (24.5)
At home	152 (29.6)	139 (27.7)	291 (28.6)
Employed	165 (32.1)	172 (34.3)	337 (33.2)
Other	75 (14.6)	64 (12.8)	139 (13.7)

SD: standard deviation.

^a^ One participant did not provide a response in the nurses’ group.

Note: For some characteristics the percentage does not add up to 100 due to rounding.

In the nurses’ group we excluded 12 women who required a β-hCG fraction test to confirm pregnancy and 28 women who had gestational durations longer than 70 days based on abdominal ultrasound examination. One woman was excluded because she changed her mind after randomization and requested manual vacuum aspiration. In the physicians’ group, we excluded two women who decided to continue with the pregnancy, 11 who required a β-hCG fraction test and 28 who had gestational durations longer than 70 days based on abdominal ultrasound examination. One woman was excluded when opting for manual vacuum aspiration after randomization and one woman was not pregnant. The number of women excluded and reasons for exclusion were similar in both groups (Fig. 1).

Forty-nine women did not return for follow-up. Attrition was similar in both groups; 6.1% (28) for nurses and 4.5% (21) for physicians (Fig. 1). The characteristics of the women lost to follow-up were similar to those who remained; however, they were more likely to be married or cohabiting (46.9% [23/49] versus 39.5% [349/884]) and be employed (42.9% [21/49] versus 31.8% [281/884]). For the analyses, 434 women remained in the nurses’ group and 450 in the physicians’ group.

Women had on average a gestational duration of 53 days by last menstrual period and 50 days by ultrasound examination. There was no significant difference in gestational duration assessed by last menstrual period or ultrasound between providers (Table 2).

**Table 2:** Assessment of gestational duration of women undergoing medical abortion provided by nurses or physicians in Mexico City, 2012–2013

Characteristic	Physicians’ group (*n* = 514)	Nurses’ group (*n* = 503)	Total (*n* = 1017)	*P*
No. of women reporting last menstrual period, (%)	464 (90.3)	451 (89.7)	915 (90)	
Duration of gestation from last menstrual period in days, mean (SD)	51.8 (14)	53.2 (16.8)	52.5 (16.1)	0.136
No. of women with ultrasound assessment, (%)	501 (97.5)	489 (97.2)	990 (97.3)	
Duration of gestation determined by ultrasound in days, mean (SD)	49.7 (13.3)	49.7 (14)	49.7 (13.3)	0.914

SD: standard deviation.

Successful medical abortions, without need for surgical intervention, were 97.9% (425/434) for nurses and 98.4% (443/450) for physicians – a result within our 5% a priori non-inferiority limit (Table 3). In 14 cases, abortion was completed after manual vacuum aspiration – nine in the nurses’ group and five in the physicians’ group. Four of the women who eventually underwent manual vacuum aspiration had initially received an additional buccal misoprostol dose at home, but at the second follow-up three still had embryonic tissue and one persistent gestational sac. The analysis also showed that providers administered an additional misoprostol dose in 68 cases. Nurses were significantly more likely than physicians to administer an extra dose (45 doses versus 23 doses; *P* < 0.05). At one facility, nurses and physicians were more likely to administer an extra dose compared with providers at the other facilities (43 doses in the nurses’ group *P* < 0.05; 17 doses in the physicians’ group *P* = 0.056).

**Table 3:** Outcomes of medical abortion provided by nurses or physicians in Mexico City, 2012–2013

Analysis	Physicians’ group	Nurses’ group	Difference, % (95% CI)
**Intention-to-treat**			
No. of women	450	434	–
Complete abortion, no. (%)^a^	443 (98.4)	425 (97.9)	0.5 (−1.2 to 2.3)
**Per-protocol**			
No. of women	406	395	–
Complete abortion, no. (%)^a^	401 (98.8)	386 (97.7)	1.0 (−0.8 to 2.9)

CI: confidence interval.

^a^ Without requiring surgical intervention.

We conducted pre-protocol analysis excluding women with the following protocol violations: taking misoprostol later than 24 hours following mifepristone administration, returning to the clinic in less than 7 days or later than 15 days after their first visit or women not having an ultrasound examination. For the analysis, 395 women remained in the nurses’ group and 406 women in the physicians’ group (Fig. 1). The results of successful medical abortion were similar to the intention-to-treat analysis (97.7% [386/395] for nurses and 98.8% [401/406] for physicians; Table 3).

We conducted a sensitivity analysis to assess the robustness of our conclusion of non-inferiority among nurses providing medical abortion in relation to physicians.^27^ We calculated two scenarios where we hypothesized the outcome for the women lost to follow-up and our results demonstrated that nurses were not inferior to physicians when providing medical abortion (Table 4).

**Table 4:** Sensitivity analysis of medical abortion provided by nurses or physicians in Mexico City, 2012–2013

Scenario^a^	Physicians’ group			Nurses’ group		Difference, % (95% CI)
	*N*	No. (%) completed abortion^b^		*N*	No. (%) completed abortion^b^	
**Scenario 1**						
Treated women without observed outcome	21	21 (100.0)		28	22 (78.6)	NA
All treated women	471	464 (98.5)		462	447 (96.8)	1.8 (−0.4 to 3.9)
**Scenario 2**						
Treated women without observed outcome	21	21 (100.0)		28	20 (71.4)	NA
All treated women	471	464 (98.5)		462	445 (96.3)	2.2 (−0.1 to 4.4)

CI: confidence interval; NA: not applicable.

^a^ Based on observed success rates, we assumed a 100% success rate for women lost to follow-up in both scenarios in the physicians’ group. For the nurses’ group we assumed 78.6% and 71.4% success rates in the first and second scenario, respectively.

^b^ Without the need for surgical intervention.

There was no difference between physicians and nurses in post-abortion contraceptive counselling and method chosen by the women. Eight-hundred and seventy-four (98.9%) women requested and were prescribed a method and 97.0% (848/874) of these women left the clinic with at least one method (Table 5). Physicians were more likely to prescribe IUDs (312 in the physicians’ group versus 267 in the nurses’ group) and women seen by physicians were more likely to leave with an IUD (135 in physicians’ group versus 100 in nurses’ group). Women treated by nurses were more likely to leave with condoms and emergency contraception, 46 in physicians’ group versus 80 in nurses’ group and 0 in physicians’ group versus 9 in nurses’ group, respectively.

**Table 5:** Contraceptive method prescribed for women undergoing medical abortion provided by nurses or physicians in Mexico City, 2012–2013

Contraceptive method	No. (%)			*P*^a^
	Physicians’ group (*n* = 450)	Nurses’ group (*n* = 434)	Total (*n* = 884)	
**No. of women prescribed contraceptives^b,c^**	444 (98.7)	430 (99.1)	874 (98.9)	ND
**Type of contraceptive prescribed^d^**				
Contraceptive injection	326 (73.4)	321 (74.7)	647 (74.0)	0.634
Intrauterine device	312 (70.3)	267 (62.1)	579 (66.2)	0.014
Oral contraceptives (estrogen and progestin pills)	296 (66.7)	267 (62.1)	563 (64.4)	0.171
Condom	255 (57.4)	256 (59.5)	511 (58.5)	0.554
Patch	138 (31.1)	147 (34.2)	285 (32.6)	0.315
Implant	118 (26.6)	123 (28.6)	241 (27.6)	0.488
Minipill (progestin only pills)	26 (5.9)	26 (6.0)	52 (5.9)	0.898
Vasectomy	19 (4.3)	22 (5.1)	41 (4.7)	0.548
Female sterilization (tubal ligation)	16 (3.6)	22 (5.1)	38 (4.3)	0.276
**No. of women leaving facility with at least one contraceptive method^c^**	432 (97.3)	416 (96.7)	848 (97.0)	ND
**Type of contraceptive taken^e^**				
Contraceptive injection	152 (35.3)	143 (34.4)	295 (34.8)	0.785
Intrauterine device	135 (31.3)	100 (24.0)	235 (27.7)	0.018
Oral contraceptives (estrogen and progestin pills)	68 (15.7)	55 (13.2)	123 (14.5)	0.291
Condom	46 (10.6)	80 (19.2)	126 (14.9)	0.000
Patch	21 (4.9)	21 (5.0)	42 (5.0)	0.906
Implant	10 (2.3)	19 (4.6)	29 (3.4)	0.072
Female sterilization (tubal ligation)	1 (0.2)	0 (0.0)	1 (0.1)	ND
Hormonal emergency contraceptive	0 (0.0)	9 (2.2)	9 (1.1)	ND

ND: not determined.

^a^ *P*-values were calculated using *χ*^2^ tests.

^b^ Contraceptive counselling took place at first visit.

^c^ Information obtained from the acceptability survey.

^d^ We had no information on the number of women prescribed hormonal emergency contraceptives.

^e^ We had no information on the number of women who chose vasectomies for their partners or minipills.

Consultation with an experienced obstetrician was done in 11 cases. Nurses consulted in six cases for administering an additional misoprostol dose, in two cases for manual vacuum aspiration and one case for a persistent gestational sac. Physicians consulted the obstetrician for interpreting ultrasound results in two cases.

On average, participants reported an acceptability score of 13.6/14 for both providers. Women in both groups reported feeling comfortable with their assigned provider (99.0% [430/434] for nurses and 98.7% [444/450] for physicians). Most women (685/884) treated by either provider reported feeling very satisfied with their service (Table 6).

**Table 6:** Acceptability survey of women undergoing medical abortion provided by nurses or physicians in Mexico City, 2012–2013

Question^a^	No. (%)		
	Physicians’ group (*n* = 450)	Nurses’ group (*n* = 434)	Total (*n* = 884)
**Did the provider explain the procedure in a clear and easy way?**			
Yes	448 (99.6)	434 (100.0)	882 (99.8)
No	2 (0.4)	0 (0.0)	2 (0.2)
**Did the provider give you time to ask questions about the procedure?**			
Yes	449 (99.8)	431 (99.3)	880 (99.5)
No	1 (0.2)	3 (0.7)	4 (0.5)
**Did the provider discuss the symptoms you may experience during the procedure?**			
Yes	447 (99.3)	433 (99.8)	880 (99.5)
No	3 (0.7)	1 (0.2)	4 (0.5)
**Did the provider discuss the warning signs that may occur during the procedure?**			
Yes	447 (99.3)	433 (99.8)	880 (99.5)
No	3 (0.7)	1 (0.2)	4 (0.5)
**Did the provider discuss the return of fertility after the medical abortion procedure?**			
Yes	395 (87.8)	394 (90.8)	789 (89.3)
No	55 (12.2)	40 (9.2)	95 (10.7)
**Did the provider take action to manage your pain?**			
Yes	449 (99.8)	434 (100.0)	883 (99.9)
No	1 (0.2)	0 (0.0)	1 (0.1)
**Could the provider have done more to control your pain?**			
Could have done more	53 (11.8)	43 (9.9)	96 (10.9)
Did enough	366 (81.3)	348 (80.2)	714 (80.8)
I did not experience pain during the procedure	31 (6.9)	43 (9.9)	74 (8.4)
**Did you have confidence in the technical skills of the provider**			
Yes	446 (99.1)	427 (98.4)	873 (98.8)
Sometimes	4 (0.9)	4 (0.9)	8 (0.9)
No	0 (0.0)	3 (0.7)	3 (0.3)
**Did the provider make you feel comfortable?**			
Yes	444 (98.7)	430 (99.1)	874 (98.9)
Sometimes	6 (1.3)	4 (0.9)	10 (1.1)
**How satisfied are you with the provider?**			
Very satisfied	342 (76.0)	343 (79.0)	685 (77.5)
Satisfied	106 (23.6)	90 (20.7)	196 (22.2)
Dissatisfied	1 (0.2)	1 (0.2)	2 (0.2)
No opinion	1 (0.2)	0 (0.0)	1 (0.1)
**Would you recommend your type of provider to a friend if she needed the same procedure?**			
Yes	444 (98.7)	427 (98.4)	871 (98.5)
Maybe	5 (1.1)	7 (1.6)	12 (1.4)
No	1 (0.2)	0 (0.0)	1 (0.1)
**How was the medical care you received from the provider in this health centre or hospital?**			
Better than you expected	431 (95.8)	408 (94.0)	839 (94.9)
As you expected	19 (4.2)	25 (5.8)	44 (5.0)
Do not know	0 (0.0)	1 (0.2)	1 (0.1)

^a^ Information regarding type of contraceptive methods prescribed and taken is presented in Table 5.

Note: For some questions the percentage does not add up to 100 due to rounding

Only one serious adverse event was recorded; a 26-year old woman at eight weeks’ gestation randomized to receive care from a physician was hospitalized for 38 hours due to bleeding following misoprostol administration and underwent a surgical abortion under general anaesthesia without further complications.

## Discussion

Our findings suggest that nurses were equal to physicians when providing medical abortion. Nurses were trained in ultrasound techniques, interpreted results and successfully managed early medical abortion up to 70 days of gestational duration as effectively as physicians. Compared with another study^11^ on the same subject, our study had higher gestational duration limits and women self-administered misoprostol at home.

The efficacy of medical abortion does not depend on who provides the medication, but on providers’ ability to correctly determine gestational duration and exclude women over 70 days’ gestation. Effective counselling for misoprostol administration at home, appropriate responses to normal and adverse effects and correct clinical decisions during follow-up are also needed. In our study, nurses were twice as likely to prescribe an additional misoprostol dose. This may reflect differences in judgment of abortion completion. Nurses may have been less confident of their skills and therefore may have depended more on ultrasound findings of persistent tissue, which is not always a sign of an incomplete abortion.^28^ At one facility, both nurses and physicians were more likely to administer an extra dose compared with providers at the other facilities. This might be explained by the higher caseload at that facility. In high-volume settings, where time is often limited, providers may give an additional misoprostol dose to be on the safe side and to use this dose as a substitute for spending more time obtaining a detailed history to assess abortion completion.

The need for an adequate learning curve for new medical abortion providers to build confidence is documented and should be given careful consideration when translating these research findings to task-shifting in programme settings.^29^

The Mexico City Ministry of Health’s guidelines mandate the use of ultrasound to determine gestational duration. While not an explicit objective determination, our findings suggest that where ultrasound is used for pregnancy dating and assessing abortion completion, nurses can manage this skill as well as physicians. These findings support the feasibility of task shifting in Mexico City.

Participants rated the medical abortion services by physicians and nurses as highly acceptable. Moreover, both types of providers were equally effective in offering post-abortion contraceptive counselling and prescribing a method. We hypothesize that differences in methods provided is due to the fact that physicians, and not nurses, routinely fit IUDs. It is possible that nurses felt less confident counselling women about IUDs. In this setting, nurses are typically responsible for providing women with condoms and emergency contraception. Familiarity with these methods is thus a potential explanation for this difference in prescribing behaviour. Because long-acting contraceptive methods such as IUD’s are more effective in reducing the likelihood of repeat unplanned pregnancy, nurses should be trained to insert them as part of routine medical care.^30^^,^^31^

A study limitation is that both types of providers practiced in the same facilities. The ethics committee of the Mexico City Ministry of Health required nurses to practice in the same facilities as physicians and would not allow the research team to alter service delivery by separating them. However, we took the necessary steps to reduce potential contamination and limited interaction by allocating different examination rooms for each provider type. This prevented them from observing or consulting with each other, although we understand this would not have prevented them from conversing in other locations. We believe these interactions would have been infrequent and would not have affected the results. Instead, keeping the providers in the same facilities might have reduced the confounding factors, because different facilities could have had different characteristics – such as availability and type of equipment and operating procedures. Further research should investigate nurses’ ability to provide medical abortion in an environment where a back-up physician may not be available.

Our study found that nurses can manage medical abortion care safely, effectively and with a high degree of patient acceptability, which is consistent with the systematic review on non-physician provision of abortion care.^32^ Enabling nurses to manage medical abortion in public health facilities or in rural areas, where there is often unmet need and less infrastructure,^33^^,^^34^ may address the high demand for safe abortion in Mexico.
